# Cholecalciferol Use Is Associated With a Decreased Risk of Incident Morphometric Vertebral Fractures in Acromegaly

**DOI:** 10.1210/clinem/dgad493

**Published:** 2023-08-22

**Authors:** Sabrina Chiloiro, Stefano Frara, Irene Gagliardi, Antonio Bianchi, Antonella Giampietro, Margherita Medici, Agnese Allora, Luigi di Filippo, Maria Rosaria Ambrosio, Alfredo Pontecorvi, Maria Chiara Zatelli, Laura De Marinis, Andrea Giustina

**Affiliations:** Department of Endocrinology and Metabolism, Pituitary Unit, Fondazione Policlinico Universitario A. Gemelli, IRCCS, Rome 00168, Italy; Dipartimento di Medicina e Chirurgia Traslazionale, Università Cattolica del Sacro Cuore, Rome 00168, Italy; Institute of Endocrine and Metabolic Sciences, Università Vita-Salute San Raffaele, IRCCS Ospedale San Raffaele, Milan 20132, Italy; Department of Medical Sciences, Section of Endocrinology, Geriatrics & Internal Medicine, University of Ferrara, Ferrara 44121, Italy; Department of Endocrinology and Metabolism, Pituitary Unit, Fondazione Policlinico Universitario A. Gemelli, IRCCS, Rome 00168, Italy; Dipartimento di Medicina e Chirurgia Traslazionale, Università Cattolica del Sacro Cuore, Rome 00168, Italy; Department of Endocrinology and Metabolism, Pituitary Unit, Fondazione Policlinico Universitario A. Gemelli, IRCCS, Rome 00168, Italy; Dipartimento di Medicina e Chirurgia Traslazionale, Università Cattolica del Sacro Cuore, Rome 00168, Italy; Department of Medical Sciences, Section of Endocrinology, Geriatrics & Internal Medicine, University of Ferrara, Ferrara 44121, Italy; Institute of Endocrine and Metabolic Sciences, Università Vita-Salute San Raffaele, IRCCS Ospedale San Raffaele, Milan 20132, Italy; Institute of Endocrine and Metabolic Sciences, Università Vita-Salute San Raffaele, IRCCS Ospedale San Raffaele, Milan 20132, Italy; Department of Medical Sciences, Section of Endocrinology, Geriatrics & Internal Medicine, University of Ferrara, Ferrara 44121, Italy; Department of Endocrinology and Metabolism, Pituitary Unit, Fondazione Policlinico Universitario A. Gemelli, IRCCS, Rome 00168, Italy; Dipartimento di Medicina e Chirurgia Traslazionale, Università Cattolica del Sacro Cuore, Rome 00168, Italy; Department of Medical Sciences, Section of Endocrinology, Geriatrics & Internal Medicine, University of Ferrara, Ferrara 44121, Italy; Department of Endocrinology and Metabolism, Pituitary Unit, Fondazione Policlinico Universitario A. Gemelli, IRCCS, Rome 00168, Italy; Dipartimento di Medicina e Chirurgia Traslazionale, Università Cattolica del Sacro Cuore, Rome 00168, Italy; Institute of Endocrine and Metabolic Sciences, Università Vita-Salute San Raffaele, IRCCS Ospedale San Raffaele, Milan 20132, Italy

**Keywords:** growth hormone, IGF-I, acromegaly, vertebral fractures, cholecalciferol‌, vitamin D

## Abstract

**Context:**

Skeletal fragility is observed in 30% to 60% of acromegaly patients, representing an emerging complication of the disease that increases disability. Despite several studies having investigated the clinical and hormonal prognostic factors for the occurrence of vertebral fractures (VFs) in acromegaly, very few data are available on their prevention/treatment including the effect of vitamin D (VD) supplementation, which has been reported to have a fracture-protective effect in several studies in patients with osteoporosis.

**Objective:**

We aimed to investigate the role of cholecalciferol (D3) supplementation in the prevention of incident VFs (i-VFs) in acromegaly.

**Methods:**

A longitudinal, retrospective and multicenter study was performed on 61 acromegaly patients treated and untreated with D3 supplementation.

**Results:**

Twenty-six patients were treated with D3 supplementation according to clinical guidelines. The median D3 weekly dosage was 8500 IU (interquartile range [IQR]: 3900). The median duration of D3 supplementation was 94 months (IQR: 38). At last follow-up, i-VFs were diagnosed in 14 patients (23%). I-VFs were less prevalent in patients on D3 supplementation (14.3% of cases) compared to patients not treated with D3 (85.7%; *P* = .02). The final level of serum V25OH-D was significantly lower in patients who developed i-VFs (28.6 ng/mL, IQR: 4.1) compared to patients who did not develop i-VFs (34.2 ng/mL, IQR: 9.6; *P* = .05). The logistic regression confirmed the protective role of D3 supplementation on the occurrence of i-VFs (odds ratio: 0.16; 95% CI, 0.03-0.79; *P* = .01).

**Conclusion:**

It is likely that D3 supplementation could lead to a reduction in i-VFs in acromegaly.

Acromegaly, a chronic condition characterized by growth hormone (GH) and, in turn, insulin-like growth factor-1 (IGF-I) excess, is burdened by a series of systemic and metabolic comorbidities that strongly impair quality of life (QoL) and life expectancy (1). Among them, a specific acromegalic osteopathy has been discovered (2), characterized by fragility fractures associated with high bone turnover that need to be detected early, according to the most recent guidelines, since they are very frequent and related to chronic pain and reduced QoL (1).

Morphometric vertebral fractures (VFs) are an emerging landmark of skeletal fragility in the general population as well as in clinical trials. They are highly prevalent in acromegaly, being reported to affect from 30% up to 60% of patients (3) and represent an early event in disease history (4). Differently from postmenopausal osteoporosis, VFs in acromegaly are not predicted by bone mineral density (BMD) measured by dual-energy x-ray absorptiometry (DXA), but are related to biochemical activity of the disease as well as disease duration (5).

In fact, VF incidence is significantly higher in patients with active disease, previous history of prolonged active disease, or relevant delay in acromegaly diagnosis (5, 6). Indeed, VFs may occur even after disease remission, particularly if hypogonadism, a reduction in BMD and/or trabecular bone score, diabetes, and/or previous VFs are present (5, 7). However, very few specific studies on prevention/treatment of VF in acromegaly, including use of vitamin D (VD), are currently available (8, 9).

VD is a key hormone for bone health and has potentially relevant extra-skeletal effects for the prevention and treatment of different diseases. Vitamin 25(OH)-D (V25OH-D) levels below 20 ng/mL are widely accepted for the definition of hypovitaminosis D, a condition still widespread in many areas of the world, due to the progressive aging of the population and change in lifestyle with reduced sun exposure in the absence of VD food fortification (10). While signs and symptoms of VD deficiency are different according to the age of individuals and may be absent in the affected population, hypovitaminosis D is recognized as the leading risk for rickets and osteomalacia. Moreover, low VD levels may lead to decreased BMD and increased fracture risk (11). Finally, despite several interventional trials in people with osteoporosis confirmed beneficial effects on skeletal health from VD supplementation, improving BMD, ameliorating muscle function, and reducing risk for fragility fractures (hip, VF, non-VF) (12-15), there is still some uncertainty as to its skeletal effects due to variable populations, doses, and type of VD used in different trials, which in a few instances have led to negative results (16-18).

The aim of the present observational study was to investigate if VD supplementation was associated with a decreased risk of incident fractures in a high-risk population such as patients with acromegaly.

## Patients and Methods

A longitudinal, retrospective, observational, and multicenter study was performed on acromegaly patients.

### Objectives

The primary objective of the study was to compare the frequency of incident VFs (i-VFs) among acromegaly patients treated and untreated with cholecalciferol (D3) supplementation.

As secondary objectives, we evaluated the correlation between the occurrence of i-VFs and serum V25OH-D concentration at last follow-up and its possible dependency on other known relevant risk factors for fractures in acromegaly, such as sex, age at acromegaly diagnosis, serum GH and IGF-I levels at diagnosis and at last follow-up, prevalent VFs, glucose metabolism abnormalities, body mass index (BMI), hypopituitarism, gonadal function and replacement daily doses of hydrocortisone (or equivalent), acromegaly outcome (disease activity), and duration of biochemical active disease.

### Inclusion/Exclusion Criteria

Acromegaly patients were consecutively enrolled from July 1, 2021 to June 30, 2022, according to the following inclusion and exclusion criteria.

Enrollment criteria included the following:

1. ascertained diagnosis of acromegaly, conducted between January 2013 to July 2020;
2. age older than 18 years;
3. patients attending the pituitary clinic of the Gemelli Hospital IRCCS, Università Cattolica del Sacro Cuore in Rome, the Endocrinological center of the Azienda Ospedaliera Universitaria of Ferrara, and IRCCS Ospedale San Raffaele in Milan;
4. data availability related to the study;
5. at least 12 months’ follow-up with either active or controlled disease.

The exclusion criteria were the following:

1. diagnosis of active neoplasia;
2. diagnosis of primary hyperparathyroidism and multiple endocrine neoplasia type 1 syndrome;
3. untreated hyperthyroidism;
4. previous or current use of bone-active drugs, including calcium. Among those with prevalent VFs, in the absence of specific guidelines and Italian drug agency recommendations for acromegaly osteopathy and due to the very little published evidence of efficacy and safety in this setting for any antiosteoporotic drugs other than bisphosphonates (8), fractured patients entering the study were mainly previously offered treatment with oral bisphosphonates. In detail, 5 of them had low (<80%) compliance to oral bisphosphonates in the previous 6 months and were discontinued, 4 refused oral bisphosphonates, 3 were intolerant to oral bisphosphonates due to gastroesophageal discomfort and denosumab was subsequently refused as patients were already under multiple-injection therapy, and 2 had contraindications to oral bisphosphonates as they were in premenopausal condition;
5. previous or current treatment with drugs known to cause fragility fractures (19) with the exception of glucocorticoid replacement therapy for central adrenal insufficiency;
6. history of spine surgery or trauma.

### Study Protocol

According to the retrospective design of the study, the baseline time point was defined as the day before the first dose of D3 supplementation in treated patients and as the day of clinical evaluation with availability of all the previously mentioned data in D3 untreated patients. D3 supplementation was prescribed and monitored in each endocrine unit according to the guidelines for treatment of VD deficiency (10) and the Italian reimbursement criteria in force during the study period. In particular, D3 was administered if serum V25OH-D was under the 20 ng/mL threshold (20) in patients considered at high risk of hypovitaminosis D due to lifestyle and clinical conditions. Dose titration of D3 was performed on the basis of periodic assessment of V25OH-D levels according to current guidelines (21, 22). Consistently with current guideline recommendations, follow-up visits were performed every 3 to 6 months for patients with active disease and every 6 to 12 months for those with controlled acromegaly.

The end of follow-up was concomitant with the last visit with availability of all clinical data on acromegaly and bone health required by the study protocol.

### Data Collection

Data on diagnosis, treatment, and clinical outcomes of acromegaly and bone health were collected. The following data were stored in an ad hoc data set: age at acromegaly diagnosis, sex, serum GH and IGF-I levels at acromegaly diagnosis, treatments for acromegaly, hypopituitarism, use and dose of replacement therapy for central adrenal insufficiency and gonadal function. Concerning bone health, the following data were collected at baseline: serum V25OH-D concentration (Roche Cobas 8000 WKC/MET/036 using electrochemiluminescence immunoassays (ECLIA); ng/mL; coefficient of variation 5%), diagnosis of osteoporosis/osteopenia and presence of VFs (prevalent VFs).

At follow-up evaluation, we collected data on serum GH and IGF-I levels, therapies for acromegaly, hypopituitarism, use and dose of replacement therapy for central adrenal insufficiency, gonadal function, D3 use, serum V25OH-D, and occurrence of i-VFs. Detailed DXA scan analyses were available both at baseline and at last follow-up for 30 out of 61 patients.

According to the American Diabetes Association, prediabetes was defined in patients with impaired fasting glucose and/or impaired glucose tolerance. Prediabetes was defined by serum fasting glucose of 100 mg/dL or greater and/or 140 mg/dL or greater at 120 minutes after 75-g oral glucose tolerance test (OGTT) and/or glycated hemoglobin A_1c_ between 5.8% and 6.4%. Diabetes mellitus was diagnosed by serum fasting glucose of 126 mg/dL or greater and/or 200 mg/dL or greater at 120 minutes after 75-g OGTT and/or glycated hemoglobin A_1c_ of 6.5% or greater (23).

### Diagnosis of Acromegaly

Based on IGF-I greater than the sex- and age-adjusted upper limit of normal (ULN) and lack of GH suppression after 75-g OGTT, acromegaly was diagnosed according to guidelines available at the time of the patients' first observation. The clinical diagnosis of acromegaly was confirmed through the histopathological examination of the pituitary tumor in those patients that underwent a neurosurgical removal of the tumor.

### Definition of Cured, Controlled, and Active Acromegaly

During the follow-up, patients underwent evaluation of GH and IGF-I levels to define disease control. Acromegaly status was defined as follows:

- cured in patients off therapy for at least 6 consecutive months, with IGF-I values below ULN, random/integrate GH below 1.0 ng/mL, and GH nadir less than 0.4 ng/mL during OGTT in nondiabetic patients;
- controlled in patients treated with medical therapy, with IGF-I values below ULN and random GH below 1.0 ng/mL (24);
- active in patients treated with medical therapy, IGF-I concentrations above ULN, and random GH higher than 1.0 ng/mL.

GH was not evaluated in patients on pegvisomant treatment (25).

IGF-I levels were expressed as a ratio to ULN, based on the normative data for each center laboratory.

According to the acromegaly consensus statements (25), during follow-up, IGF-I reflects the clinical activity of the disease, whereas random serum GH the presence of adenomatous tissue. In patients with discrepant levels of GH and IGF-I, the consensus recommends relying on IGF-I values, after ensuring the use of a well-validated IGF-I assay and after ruling out preanalytic and analytical confounding factors (25) such as malnutrition, obesity, eating disorders, poorly controlled diabetes mellitus, cystic fibrosis, hepatic and renal disease, hypothyroidism, hyperthyroidism, sepsis, and testosterone overtreatment (26).

### Evaluation of Vertebral Fractures

As per good clinical practice in our pituitary units and according to 2013 guidelines on the management of comorbidities in acromegaly (27), vertebral morphometry on dorso-lumbar spine x-ray was conducted at the time of acromegaly diagnosis and every 2 years, during follow-up.

Using a semiquantitative morphometric approach (3-5), anterior (Ha), middle (Hm), and posterior (Hp) vertebral heights were measured, and height ratios were calculated for each vertebra from T4 to L4. Prevalent VFs were identified on the basal radiographs, whereas i-VFs were identified on spinal radiographs during follow-up but absent at baseline. According to Genant's classification (28), VFs were defined as mild, moderate, or severe if a decrease in Ha/Hp, Hm/Hp, or Hp/Hp of the adjacent vertebrae ratio was found of 20% to 25%, 25% to 40% or greater than 40%, respectively.

### Statistical Analysis

The patient cohort was described in its clinical and demographic features using descriptive statistics techniques. Normality of continuous variables was checked using the Kolmogorov-Smirnov test. Quantitative variables were expressed as median and range and qualitative variables as absolute and percentage frequency. Chi-square test (or Fisher exact test when necessary) and Mann-Whitney nonparametric tests were used to compare categorical and quantitative unpaired data. In the non-dichotomic categorical variables including more than two subgroups, differences of percentages between specific subgroups were analysed through Fisher exact test using as comparator the group with the highest patient’s number (identified as reference group). Receiver operating characteristic curves were constructed to assess the ability of GH and V25OH-D levels to discriminate between patients with and without i-VFs. The variables that reached statistical significance at the univariate analysis entered the logistic regression and were adjusted for age and sex. The analyses were performed using SPSS software version 24.0 for Windows.

### Ethical Approval

All procedures performed in the study were in accordance with the ethical standards of the institutional review board and with the 1964 Helsinki Declaration and its later amendments or comparable ethical standards. The study was approved by local institutional review boards. All patients signed an informed consent before entering the study.

## Results

### Baseline Data

#### Whole population

A total of 61 patients entered the study. Thirty-seven were female (60.7%) and 24 were male (39.3%). At acromegaly diagnosis, median age was 43 years (interquartile range [IQR]: 18 years), median serum GH 11.8 ng/mL (IQR: 13), and median IGF-I was 2.75× ULN (IQR: 3.25). Ten patients (16.4%) harbored a microadenoma, whereas 51 (83.6%) had a macroadenoma. Partial hypopituitarism occurred in 27 (44.3%) patients. Twenty-eight (45.9%) patients were affected by central adrenal insufficiency: 19 were treated with no more than 20 mg hydrocortisone daily and 9 patients with more than 20 mg daily.

Median BMI in the whole cohort was 29.9 (IQR: 5.1). Twenty-nine patients were affected by prediabetes or diabetes (47.5%).

At baseline, the median serum V25OH-D level was 19.5 ng/mL (IQR: 1.3) in the whole study population. Fourteen patients (23%) carried prevalent VFs. The median serum V25OH-D concentration was 21.9 (IQR: 8) ng/mL in patients without prevalent VFs and was 19.5 (IQR: 9.4) ng/mL in patients with prevalent VFs (*P* = .825). The basal serum concentration of V25OH-D did not correlate with IGF-I (*P* = .174; *r*: 0.667), while nonsignificant trends toward V25OH-D and spine (*P* = .09, *r*: 0.345) and femoral neck (*P* = .06, *r*: 0.386) T-scores were observed.

Serum V25OH-D levels were significantly lower in patients on treatment with somatostatin analogues (SSAs) (median V25OH-D 27.1 ng/mL IQR: 29.4) compared to patients not treated with SSAs (median 32 ng/mL IQR: 5.7; *P* < .001).

#### Patients treated vs untreated with cholecalciferol

Twenty-six patients (42.6%) were chronically supplemented with D3: 12 patients were treated with first-generation SSAs (46.2%), 8 with pegvisomant (30.8%), a single patient with pasireotide Lar (3.8%), and 2 with combination of pasireotide Lar and pegvisomant (7.7%). The remaining 3 patients were off therapy and consequently considered with cured acromegaly (11.5%). All patients treated with D3 had a serum V25OH-D concentration lower than 20 ng/mL before starting supplementation, whereas all patients not treated with D3 had V25OH-D higher than 20 ng/mL, except for 7 who refused or were noncompliant with D3 supplementation.

Serum V25OH-D levels at baseline were slightly but not significantly lower in D3 treated vs untreated patients (18 IQR: 2 vs 21.9 IQR: 1 ng/mL; *P* = .152) (Table 1).

**Table 1.** Clinical and biochemical features of patients treated and untreated with D3 supplementation at baseline

	Whole study cohort	Patients not on D3 supplementation	Patients on D3 supplementation	*P*
No. of patients	61	35	26	
Age at acromegaly diagnosis, median yrs (IQR)	43 (18)	42.4 (15)	43.9 (15.6)	.634
Sex				.237
Female, n (%)	37 (60.7%)	19 (54.3%)	18 (69.2%)	
Male, n (%)	24 (39.3%)	16 (45.7%)	8 (30.8%)	
BMI, median (IQR)	29.9 (5.1)	29.1 (4.8)	30.2 (8.5)	.725
GH at acromegaly diagnosis, median ng/mL (IQR)	11.8 (13)	12.5 (9.5)	11.3 (5.8)	.757
IGF-I × ULN at acromegaly diagnosis, median (IQR)	2.75 (3.25)	2.7 (0.89)	3.4 (0.75)	.1
Diagnosis of osteoporosis/osteopenia at baseline				
Yes, n (%)	12 (19.7%)	6 (17.1%)	6 (23.1%)	.564
No, n (%)	49 (80.3%)	29 (82.9%)	20 (76.9%)	
Diagnosis of prediabetes/diabetes mellitus				
Yes, n (%)	29 (47.5%)	15 (42.9%)	14 (53.8%)	.395
No, n (%)	32 (52.5%)	20 (57.1%)	12 (46.2%)	
Prevalent VFs				
Yes, n (%)	14 (23%)	10 (28.6%)	4 (15.4%)	.226
No, n (%)	47 (77%)	22 (71.4%)	25 (84.6%)	
Hypopituitarism				
Yes, n (%)	27 (44.3%)	14 (40%)	13 (50%)	.437
No, n (%)	34 (55.7%)	21 (60%)	13 (50%)	
Serum V25OH-D at baseline, median ng/mL (IQR)	19.5 (1.3)	21.9 (1)	18 (2)	.152
Replacement therapy for central adrenal insufficiency				
None, n (%)*	33 (54.1%)	18 (51.4%)	15 (57.7%)	
Daily dose <20 mg/d, n (%)	19 (31.1%)	13 (37.1%)	6 (23.1%)	.409
Daily dose >20 mg/d, n (%)	9 (14.8%)	4 (11.4%)	5 (19.2%)	.329
Gonadal function				
Eugonadal patients, n (%)*	40 (65.6%)	26 (76.5%)	14 (77.8%)	
Hypogonadal patients, n (%)	3 (4.9%)	2 (5.9%)	1 (5.6%)	.953
Menopausal women, n (%)	9 (14.8%)	6 (17.6%)	3 (16.7%)	.924
Acromegaly therapy at baseline				
First-generation SSAs, n (%)*	26 (42.6%)	14 (40%)	12 (46.2%)	
Observation, n (%)	13 (21.3%)	10 (28.6%)	3 (11.5%)	.163
Pegvisomant, n (%)	17 (27.9%)	9 (25.7%)	8 (30.8%)	.177
Pasireotide Lar, n (%)	2 (3.3%)	1 (2.9%)	1 (3.8%)	.476
Pasireotide Lar + pegvisomant, n (%)	3 (4.9%)	1 (2.9%)	2 (7.7%)	.142

Univariate analysis.

Abbreviations: BMI, body mass index; D3, cholecalciferol; GH, growth hormone; IQR, interquartile range; Ref, reference; SSA, somatostatin analogue; ULN, upper limit of normal; V25OH-D, vitamin 25(OH)-D; VF, vertebral fracture; yrs, years.

*Reference group for statistical analysis. Values in round brackets are IQR; percentage in round brackets are referred to the total number of patients of each table column.

As shown in Table 1, at baseline the frequencies of osteoporosis/osteopenia and prevalent VFs were similar among the groups of patients subsequently treated or untreated with D3 supplementation. The two groups did not differ by age at acromegaly diagnosis, sex, BMI, GH, and IGF-I levels at acromegaly diagnosis, prevalence of hypopituitarism, prediabetes/diabetes mellitus, or central adrenal insufficiency, daily dose of hydrocortisone (or equivalent), prevalence of central hypogonadism or menopause, and class of drug for acromegaly treatment.

### Follow-up Data

The median D3 weekly dosage at last follow-up was 8500 IU (IQR: 3900 IU). The median duration of D3 supplementation was 94 months (IQR: 38 months). No adverse events related to D3 supplementation were reported by patients or identified from medical records. At last follow-up, serum V25OH-D was significantly higher in patients on D3 supplementation compared to untreated ones (40.9 IQR: 8.1 vs 28.1 IQR: 4.8 ng/mL; *P* < .001) (Table 2).

**Table 2.** Biochemical and densitometric (*^a^*, in 30 patients only) features of patients treated and untreated with D3 supplementation at last follow-up

	Whole study cohort	Patients not on D3 supplementation	Patients on D3 supplementation	*P*
Spine T-score at last FUP, median (IQR)*^a^*	−1.1 (1.4)	−1.15 (1.4)	−0.9 (1.5)	.998
Δ spine T-score last FUP vs baseline, median (IQR)*^a^*	−0.3 (0.5)	−0.1 (0.7)	−0.75 (1.2)	.102
Femoral neck T-score at last FUP, median (IQR)*^a^*	−0.5 (1.3)	−0.55 (1.25)	−0.5 (1.7)	.925
Δ femoral neck T-score last FUP vs baseline, median (IQR)*^a^*	−0.3 (0.9)	−0.1 (1.1)	−0.5 (2)	.856
Serum V25OH-D at last FUP, median ng/mL (IQR)	30.2 (13.8)	28.1 (4.8)	40.9 (8.1)	**<.001**

Univariate analysis.

Abbreviations: D3, cholecalciferol; FUP, follow-up; IQR, interquartile range; V25OH-D, vitamin 25(OH)-D.

*P* values in bold are statistically significant.

Serum V25OH-D level was significantly higher (median 31.2 ng/mL; IQR: 11.9 ng/mL) in patients with controlled acromegaly compared to the serum V25OH-D level (median 25 ng/mL IQR: 2 ng/mL; *P* = .038) in patients with active acromegaly. The variation (Δ) of serum V25OH-D level from baseline to last follow-up (1.5; IQR: 0.5) was significantly higher in acromegaly patients who reached the biochemical control, compared to those with active disease at last follow-up (1.3 IQR: 0.2; *P* < .001).

At last follow-up, i-VFs were diagnosed in 14 patients (23% of cases): 2 patients were on treatment with D3 supplementation (14.3%) and 12 patients were not on D3 supplementation (85.7%; *P* = .02) (Table 3); among this latter group (patients not treated with D3 with i-VFs), 6 had no compliance and withdrew D3 after 1 to 3 months, 3 had low compliance with the D3 regimen and withdrew it after 3 to 6 months, and 3 refused therapy.

**Table 3.** Clinical and biochemical features of patients with and without incident vertebral fractures at baseline

	Incident VFs		*P*
	No	Yes	
No. of patients (%)	47	14	
Age at acromegaly diagnosis, median y (IQR)	45 (11.6)	30 (7.5)	.22
Sex			
Female, n (%)	29 (61.7%)	8 (57.1%)	.759
Male, n (%)	18 (38.3%)	6 (42.9%)	
GH at acromegaly diagnosis, median ng/mL (IQR)	10.3 (6.5)	21.8 (10.2)	.**01**
IGF-I × ULN at acromegaly diagnosis, median (IQR)	2.9 (0.8)	3.7 (0.5)	.19
Disease activity at baseline			
Cured/Controlled, n (%)	38 (80.9%)	8 (57.1%)	.07
Active, n (%)	9 (19.1%)	6 (42.9%)	
Prevalent VFs			
Yes, n (%)	6 (12.8%)	8 (57.1%)	.**001**
No, n (%)	41 (87.2%)	6 (42.9%)	
Patients on D3 supplementation			
Yes, n (%)	24 (51.1%)	2 (14.3%)	.**02**
No, n (%)	23 (48.9%)	12 (85.7%)	
Diagnosis of prediabetes/diabetes mellitus			
Yes, n (%)	25 (53.2%)	7 (50%)	.824
No, n (%)	22 (46.8%)	7 (50%)	
Hypopituitarism			
Yes, n (%)	19 (40.4%)	8 (57.1%)	.269
No, n (%)	28 (59.6%)	6 (42.9%)	
Gonadal function			
Eugonadal patients, n (%)*	33 (86.8%)	7 (50%)	
Hypogonadal patients, n (%)	2 (5.3%)	1 (7.1%)	.47
Menopausal women, n (%)	3 (7.9%)	6 (42.9%)	.06
Replacement therapy for central adrenal insufficiency			
None, n (%)*	27 (57.4%)	6 (42.9%)	
Daily dose <20 mg/d, n (%)	11 (23.4%)	8 (57.1%)	.06
Daily dose >20 mg/d, n (%)	9 (19.1%)	0 (0%)	.167

Univariate analysis.

Abbreviations: D3, cholecalciferol; GH, growth hormone; IGF-I, insulin-like growth factor-1; IQR, interquartile range; Ref, reference; ULN, upper limit of normal; VF, vertebral fracture.

*P* values in bold are statistically significant.

*Reference group for statistical analysis. Values in round brackets are IQR; percentage in round brackets are referred to the total number of patients of each table column.

**Figure 1.**
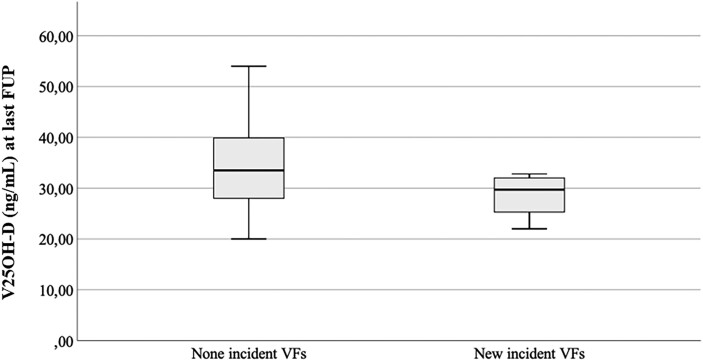
Serum vitamin 25OH-D (V25OH-D) levels at last follow-up (FUP) in acromegaly patients with and without incident vertebral fractures (VFs). Univariate analysis (*P* = .05).

The final level of serum V25OH-D was significantly lower in patients who developed i-VFs (28.6 ng/mL, IQR: 4.1 ng/mL) compared to patients who did not develop i-VFs (34.2 ng/mL, IQR 9.6 ng/mL; *P* = .05) (Fig. 1 and Table 4). No difference was observed in terms of BMI for patients who experienced i-VFs compared to those without i-VFs (28.4 [IQR: 3.9] vs 31.4 [IQR: 7.2]; *P* = .148), and there was no significant correlation between BMI and V25OH-D serum levels at last follow-up (*P* = .25; *r*: −0.21) or ΔV25OH-D (*P* = .36; *r*: −0.19).

**Table 4.** Clinical, biochemical and densitometric (*^a^*, in 30 patients only) features of patients with and without incident vertebral fractures at last follow-up

	Incident VFs		*P*
	No (n.47)	Yes (n.14)	
Acromegaly at FUP			
Cured/Controlled, n (%)	40 (85.1%)	13 (92.9%)	.451
Active, n (%)	7 (14.9%)	1 (7.1%)	
Active disease duration, median mo (QR)	13 (15)	14 (10)	.58
GH at last follow-up, median ng/mL (IQR)	1.9 (1.9)	3 (2.7)	.39
IGF-I × ULN at last follow-up, median (IQR)	0.8 (0.5)	0.8 (0.4)	.922
Spine T-score at last FUP, median (IQR)*^a^*	−1.1 (2.8)	−1.2 (1.5)	.09
Δ spine T-score last FUP vs baseline, median (IQR)*^a^*	−0.4 (1.7)	−0.7 (2.1)	.07
Femoral neck T-score at last FUP, median (IQR)*^a^*	−0.4 (1.5)	−0.1 (0.9)	.7
Δ femoral neck T-score last FUP vs baseline, median (IQR)*^a^*	−0.5 (2.1)	0.2 (1.5)	.51
Serum V25OH-D ng/mL (FUP), median (IQR)	34.2 (9.6)	28.6 (4.1)	**.05**

Univariate analysis.

Abbreviations: D3, cholecalciferol; FUP, follow-up; GH, growth hormone; IGF-I, insulin-like growth factor-1; IQR, interquartile range; ULN, upper limit of normal; V25OH-D, vitamin 25(OH)-D; VF, vertebral fracture.

*P* values in bold are statistically significant. Values in round brackets are IQR; percentage in round brackets are referred to the total number of patients of each table column.

**Figure 2.**
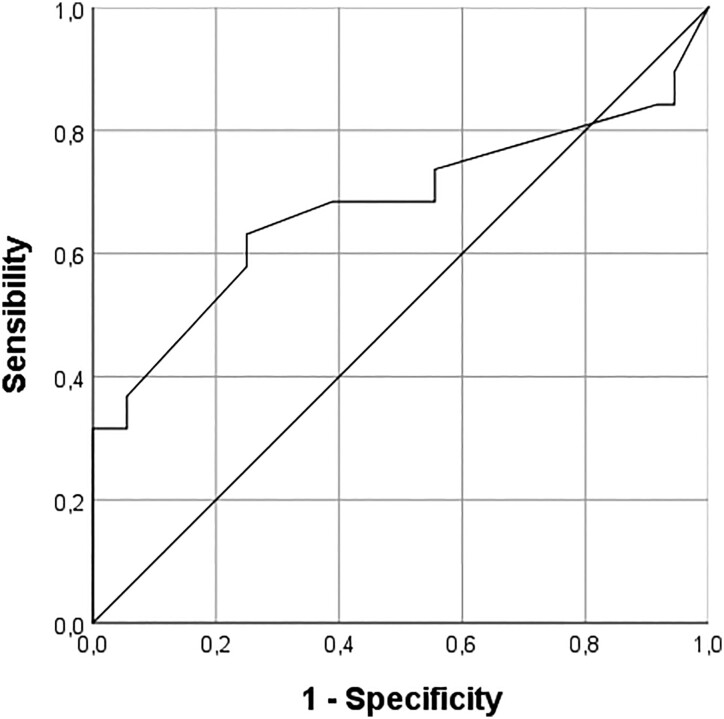
Receiver operating characteristic curve analysis: the role of serum V25OH-D levels at last follow-up in the prediction of incident vertebral fracture. The cutoff point of 31 ng/mL provided 67% sensitivity and 100% specificity (area under the curve 0.967; *P* = .013).

Receiver operating characteristic analysis was performed to determine the role of V25OH-D levels at last follow-up in the prediction of i-VF. The cutoff point of 31 ng/mL for V25OH-D provided 67% sensitivity and 100% specificity with an area under the curve equal to 0.967, a positive predictive value of 100%, and negative predictive value of 60% (*P* = .013) (Fig. 2).

Median Δ V25OH-D levels from baseline to follow-up were higher in patients without i-VFs (3 ng/mL; IQR: 7.3 ng/mL), compared to those reached in patients who did experience i-VFs (1.6 ng/mL; IQR: 0.5 ng/mL; *P* = .03).

**Figure 3.**
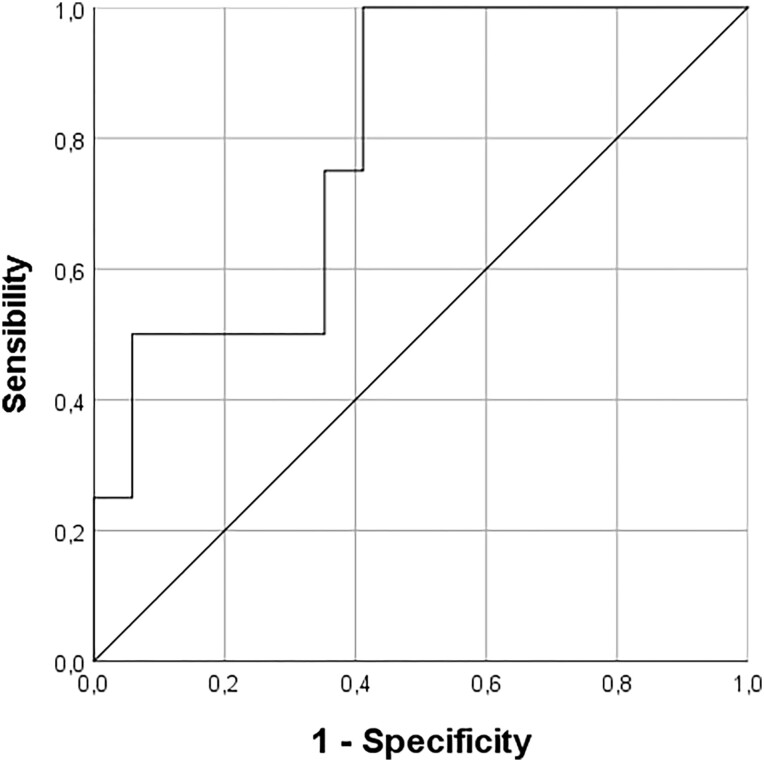
Receiver operating characteristic curve analysis: the role of serum growth hormone levels at acromegaly diagnosis in the prediction of incident vertebral fracture. The cutoff point of 12 ng/mL provided 100% sensitivity and 77% specificity (area under the curve 0.87; *P* = .04).

Moreover, patients with i-VFs had higher serum GH levels at acromegaly diagnosis (21.8 ng/mL; IQR: 10.2 ng/mL) than those who did not experience i-VFs (median GH 10.3 ng/mL; IQR: 6.5 ng/mL; *P* = .01) (see Table 3). Patients with serum GH level higher than 12 ng/mL at acromegaly diagnosis (*P* = .04; area under the curve: 0.87, sensitivity 100%, specificity 77%; positive predictive value 100%, negative predictive value 43.5%) more frequently experienced i-VFs (Fig. 3). Conversely, IGF-I levels at baseline were only marginally and nonsignificantly more elevated in patients who subsequently developed VFs vs those who did not (*P* = .19) (see Table 3). Serum GH and IGF-I, as well as glycemic status at last follow-up, were not significantly different in acromegaly patients with and without i-VFs (*P* = .39, *P* = .92, and *P* = .824, respectively). Disease control and presumed length of active disease were not different in patients with i-VFs vs without i-VFs, although it has to be noted that only 1 out of 14 newly fractured patients had active disease (see Table 4).

Patients with prevalent VFs more frequently developed i-VFs (57.1%) compared to patients without prevalent VFs (42.9%; *P* = .001). Furthermore, in the group of patients with i-VFs there was a slightly but nonsignificantly higher percentage of men than women (42.9% vs 38.3%; *P* = .759) and the prevalence of postmenopausal women among patients with i-VFs was greater vs patients without i-VFs, although this difference did not reach statistical significance (3 [7.9%] vs 6 [42.9%]; *P* = .06).

Finally, comparing patients with and without i-VF, we found no difference in spine and hip T-score levels expressed as both absolute and Δ from baselines (see Table 4).

### Predictive Factors for Incident Vertebral Fractures

At the multivariate logistic regression, supplementation with D3 resulted a protective factor for the occurrence of i-VFs (odds ratio [OR]: 0.16; 95% CI, 0.03-0.79; *P* = .01). Instead, nonsupplementation with D3 resulted in being a risk factor for the occurrence of i-VFs (OR: 4.5; 95% CI, 1.1-18.2; *P* = .01). Moreover, the presence of prevalent VFs and higher serum GH levels were found to be additional risk factors for the occurrence of i-VFs (respectively OR 9.1; 95% CI, 2.3-35.5; *P* < .001 and OR 1.5; 95% CI, 1.1-2.4; *P* = .019) (Table 5).

**Table 5.** Multiple linear regression for the occurrence on incident vertebral fractures

	*P*	OR (95% CI)
GH ng/mL at acromegaly diagnosis	.02	1.5 (1.1-2.4)
Prevalent VFs	<.001	9.1 (2.3-35.5)
D3 supplementation	.01	0.16 (0.03-0.79)
No D3 supplementation	.01	4.5 (1.1-18.2)

Abbreviations: D3, cholecalciferol; GH, growth hormone; OR, odds ratio; VF, vertebral fracture.

## Discussion

In this study, we investigated the effect of D3 supplementation on the occurrence of i-VFs in a multicentric, retrospective, and longitudinal series of acromegaly patients.

We report for the first time that D3 supplementation is associated with a reduced VF incidence in acromegaly patients. In this context, D3 was safe and effective in raising V25OH-D levels in the so-called desirable range and patients who had i-VFs had slightly but significantly lower V25OH-D levels compared to nonfractured patients. Finally, no D3 supplementation, very high GH levels at diagnosis, and/or prevalent fractures at baseline represented independent risk factors for i-VFs.

VD is a key hormone for intestinal calcium absorption and bone mineralization. Its severe deficiency causes rickets in children and osteomalacia in adults. Less severe VD deficiency is associated with an increased risk of osteoporosis and fragility fractures in the general population (29).

Current guidelines recommend D3 supplementation in patients with V25OH-D deficient levels (30). VFs are a landmark of acromegaly osteopathy as in other forms of secondary osteoporosis (9); they are not reliably predicted by BMD measured by DXA in acromegaly (31, 32), but predominantly by disease activity (specifically by GH levels) and active disease duration (5), being an early phenomenon in the natural history of the disease (4) and related to long diagnostic delay (6). However, despite osteopathy being an emerging comorbidity of acromegaly and the known role of VD in bone health, there are still limited data on VD status in individuals with acromegaly. Interestingly, VD deficiency was invariably found in patients with active disease who showed lower V25OH-D levels vs control individuals with a significantly positive correlation between IGF-I and V25OH-D levels in active patients (33). Other reports have suggested that free VD is decreased in acromegaly and that tt (TaqI), aa (ApaI), and bb (BsmI) polymorphisms of the *VDR* gene may be associated with better bone quality and microarchitecture (higher trabecular bone score), which lead to a lower risk of osteoporotic fractures in acromegaly patients (34, 35). A possible role for lipophilic substance malabsorption by SSA in determining low VD levels was hypothesized (36) but not confirmed by more recent studies (37, 38). In this cohort, we found that patients on SSA treatment had lower V25OH-D levels compared to patients not undergoing SSA treatment.

Supplementation with D3 in the general population did not result in prevention of fractures (15), whereas supplementing individuals at high risk of hypovitaminosis D, such as the geriatric population or other special groups (ie, those with postmenopausal and glucocorticoid-induced osteoporosis), may have protective effects on their high risk of fracture (15). Nevertheless, available meta-analyses still provide conflicting results on the antifracture effect of VD due to selection biases, including patients with normal levels of VD, use of different doses of D3 and combinations with calcium, or length of follow-up (16-18).

Prevention of VFs in acromegaly remains an open issue. It has been shown that use of GH/IGF-I–lowering treatments with first-generation SSAs and pegvisomant may reduce the risk of VFs while improving disease control (39, 40). Moreover, the second-generation SSA pasireotide Lar was recently shown to be more protective than pegvisomant against VF risk, possibly via a direct osteometabolic effect or via GH inhibition (41). In fact, our data show that baseline GH levels are able to predict i-VF risk, whereas disease control as assessed by IGF-I levels during observation does not significantly affect fracture risk despite uncontrolled patients having a reduced V25OH-D response to D3. This nonsignificant result may also be due, at least in part, to the very few numbers of patients that remained with active disease during follow-up (8/61). Evidence concerning the effects of specific bone-sparing treatments in acromegalic osteopathy is even more scanty. It was hypothesized that because this form of osteoporosis is characterized by increased bone turnover, antiresorptive agents could have a beneficial role. However, it was recently reported that retrospectively, only in patients enrolled with active disease, did any kind of treatment (antiresorptive with bisphosphonates or denosumab and anabolic with teriparatide) have a significant effect on i-VFs (8). In the study, most patients were on D3 and/or calcium supplementation, and no information was given about a possible role of VD supplementation on bone health (8).

Recently, in a prospective study conducted on 34 patients with active acromegaly and 30 age-, sex-, and BMI-matched control individuals, the response to a single dose (150 000 IU) of oral D3 was evaluated. Interestingly, acromegaly patients showed a lower increase in free V25OH-D levels and ΔV25OH-D was negatively correlated with disease activity as assessed by both IGF-I and fasting GH levels without correlation with BMI (42).

Therefore, none of the previous studies provided any information on the effects of long-term D3 supplementation on skeletal outcomes in patients with acromegaly. Our data show for the first time a possible protective effect of long-term D3 supplementation on VF occurrence in the high-risk setting of acromegalic osteopathy.

Our results confirm that acromegaly is a high-risk condition for VFs since around 25% of patients developed such fractures during follow-up, in line with previously reported data (9).

Interestingly in this study, the duration of biochemical active disease was shorter with respect to previous literature (39-41). This study population reflects the well-known modern management of acromegaly patients that is oriented to reduce the time of exposure to GH and IGF-I hypersecretion. The patients included in this study were diagnosed with acromegaly since 2013. According to the availability of new, efficacious, and safe treatments for acromegaly, in our real-life clinical practice patients not responsive to first-generation SSAs are switched to second-line therapies after at maximum of 12 months of treatment with first-generation SSAs. Moreover, second-line therapies are personalized according to clinical and biochemical individual paraments as well as tumor molecular and morphology features (43, 44) to optimize the acromegaly outcome and to reduce the time of exposure to GH and IGF-I hypersecretion. The persistence and long-term biochemical active disease are well recognized as risk factors for the development of acromegaly-related comorbidities that affect life expectancy and the QoL of these fragile patients (45).

The original aspect of our observation is that more than double the number of i-VFs occurred in the D3 nonsupplemented group. Moreover, fractured patients showed slightly but significantly lower circulating V25OH-D levels compared to nonfractured patients and a lack of supplementation with D3 was independently negatively associated with occurrence of i-VF. It is also worth mentioning that in our treated group desirable V25OH-D values were obtained using similar doses with respect to those recommended for the general osteoporotic population, in line with the recommended daily intake of about 1000 IU per day (30). Moreover, serum V25OH-D levels were significantly lower in patients with active disease at follow-up, confirming that active acromegaly may represent a state of relative “VD resistance,” as already reported for Cushing disease (46, 47).

Finally, D3 was prescribed at all centers following shared guidelines (30) based on the detection of V25OH-D under the 20 ng/mL threshold in patients considered at high risk of hypovitaminosis D due to lifestyle and clinical conditions (10). An ad hoc metanalysis reported that disease activity, male sex, and hypogonadism were related to VF risk in acromegaly (48). Our multiple regression analysis partly confirmed these data since GH levels and prevalent VFs at study entry were independent predictors of i-VF. In detail, the regression model proved that acromegaly patients with prevalent VFs had a 9-fold increased risk of developing i-VFs, those not D3 vitamin supplemented had a 4.5-fold increased risk for i-VFs, and those with higher serum GH levels had a 1.5-fold increased risk of i-VFs development. Conversely, a nonsignificant effect of sex and gonadal status if not a trend toward a higher prevalence of postmenopausal women in the i-VF group was observed.

One of the main limitations of our study was the absence of a standardization for V25OH-D levels. V25OH-D was measured for each patient in the same laboratory through the follow-up, the same commercial methods and laboratory thresholds were cross-sectionally applied by all centralized laboratories. Moreover, the retrospective design of the study did not allow us either to randomly assign patients into the two treatment-groups (patients treated or untreated with D3 supplementation) or to correct VD data based on the season of the year in which samples were obtained (10). In this view, a prospective and randomized study is advocated to further validate our data, although observational pilot studies have a recognized value in this clinical setting (49). In fact, due to the nature of the study, the decision to supplement or not patients with VD was based on clinical judgment that, in turn, relied on available guidelines for the general population, since no specific recommendations have been issued yet for patients with acromegaly. Nevertheless, very few patients with prevalent VFs were not initiated with D3 supplementation. In the same vein, according to the retrospective nature of this study we excluded from this patient cohort those receiving any specific antiosteoporotic treatments, as per the study design. The application of such stringent inclusion and exclusion criteria allowed us to investigate VD effects independently from the use of other bone-active drugs. Moreover, the availability of this latter subpopulation also allowed us to specifically investigate the antifracture protective effect of VD in a setting of very high VF risk, as the acromegaly population bearing prevalent VFs. On the other hand, application of such stringent entry criteria resulted in a relatively limited number of participants recruited, which, however, is also due to the rarity of the disease (50) and the still not extensive osteometabolic evaluation of all acromegaly patients, despite being clearly indicated by available guidelines on the assessment of the disease complications (1). Finally, the study was carried out in Italy, an area characterized by widespread VD deficiency (51), due to lack of food fortification with VD. Therefore, our data may not be directly translated in practice in VD-replete areas of the world.

Besides these aforementioned limitations, our study has at least two relevant clinical implications. In fact, patients with acromegaly, particularly those with biochemically active disease, are at high risk of concomitantly developing VFs and hypovitaminosis D. Due to the observed protective effect of D3 on i-VFs, our data suggest that, particularly in areas at high prevalence of hypovitaminosis, V25OH-D should be measured in all patients with acromegaly and D3 supplementation started at standard doses in those with low V25OH-D levels (52).

Since in our hands D3 supplementation appeared to also be safe in acromegaly, we suggest that guidelines will recommend this osteometabolic aspect be managed in every endocrine center, leaving the choice of specific antiosteoporotic treatments to the bone expert in the multidisciplinary team of pituitary tumor centers of excellence (53).

Moreover, the observed bone-sparing effect of D3 supplementation may have clinical relevance besides the specific setting of acromegaly. In fact, morphometric VFs have been shown to be a marker of frailty, clearly associated with impaired QoL and reduced life expectancy in postmenopausal women, but also in men and in COVID-19 patients (54, 55). Indeed, clinical diagnosis heavily underestimates the dimension of the problem, since most VFs are asymptomatic, pauci-symptomatic, or characterized by self-limited pain (56, 57). However, few studies have been able to investigate the effect of V25OH-D levels on prevalence of morphometric VFs (58, 59), with discordant results. Moreover, data on D3 supplementation on morphometric VF risk derive only from the control arm of antiosteoporotic drug trials (60, 61). Therefore, our data confirm that VD status may affect morphometric fracture prevalence, but, above all, D3 supplementation alone may be associated with reduced incidence of these fractures, at least in a high-risk population, living in a largely VD-deficient area.

In conclusion, our data suggest that VD status may have a relevant impact on morphometric fracture risk in acromegaly and should be checked in all patients with this disease. Moreover, since D3 supplementation at standard doses was associated with reduced risk of i-VF, we suggest that all VD-deficient patients with acromegaly be supplemented with D3 at standard doses.

## Data Availability

The data sets generated during and/or analyzed during the current study are not publicly available but are available from the corresponding author on reasonable request.

## Abbreviations

BMD: bone mineral density
BMI: body mass index
D3: cholecalciferol
DXA: dual-energy x-ray absorptiometry
GH: growth hormone
IQR: interquartile range
i-VF: incident vertebral fracture
OGGT: oral glucose tolerance test
OR: odds ratio
QoL: quality of life
SSA: somatostatin analogue
ULN: upper limit of normal
V25OH-D: vitamin 25(OH)-D
VD: vitamin D
VF: vertebral fracture
